# Development of Spray Dried *Spirulina* Protein-Berry Pomace Polyphenol Particles to Attenuate Pollution-Induced Skin Damage: A Convergent Food-Beauty Approach

**DOI:** 10.3390/antiox12071431

**Published:** 2023-07-15

**Authors:** Roberta Targino Hoskin, Mary H. Grace, Anna Guiotto, Alessandra Pecorelli, Giuseppe Valacchi, Mary Ann Lila

**Affiliations:** 1 Plants for Human Health Institute, Food, Bioprocessing & Nutrition Sciences, North Carolina State University, North Carolina Research Campus, Kannapolis, NC 28081, USA; rtcorrei@ncsu.edu (R.T.H.); mhgrace@ncsu.edu (M.H.G.); 2 Plants for Human Health Institute, Animal Science Department, North Carolina State University, North Carolina Research Campus, Kannapolis, NC 28081, USA; aguiott@ncsu.edu (A.G.); apecore@ncsu.edu (A.P.); 3 Department of Environmental and Prevention Sciences, University of Ferrara, 44121 Ferrara, Italy; 4 Department of Food and Nutrition, Kyung Hee University, Seoul 02447, Republic of Korea

**Keywords:** sustainability, value-added, repurposing, skin health, cosmeceuticals

## Abstract

Spray drying (SD) microencapsulation of phytochemicals from berry pomaces with *Spirulina* protein (SP) was incorporated into a cosmeceutical topical formulation to mitigate pollution skin damage. Initially, microparticles produced with SP and polyphenols recovered from fruit pomaces (elderberry SP-EB and muscadine grape SP-MG) were characterized regarding physicochemical and phytochemical content (polyphenol load, carotenoid and phycocyanin contents and antioxidant activity). SP had low total phenolic content (7.43 ± 0.23 mg GAE/g DW), but complexation with elderberry or muscadine grape pomaces polyphenols led to a substantial increase (27.63 ± 1.15 SP-EB and 111.0 ± 2.6 mg GAE/g DW SP-MG). SP-MG particles had higher anthocyanin (26.87 ± 1.25 mg/g) and proanthocyanidin (9.02 ± 0.74 mg/g) contents compared to SP-EB particles. SP-MG were prioritized to prepare a topical gel to attenuate skin oxinflammatory markers and prevent skin barrier disruption using ex vivo human biopsies exposed to diesel engine exhaust (DEE). The immunofluorescence results showed increased oxidative protein damage and inflammation associated with impaired skin barrier function after DEE exposure while topical application of gel formulated with SP-MG mitigated these effects. Overall, this study demonstrated that protein-polyphenol complexation is a synergistic strategy to stabilize and deliver residual fruit/algae phytoactives into cosmeceutical products for skin health applications.

## 1. Introduction

An estimated 4.2 million deaths globally are linked to ambient air pollution [1]. The direct relationship between air pollution exposure and increased mortality caused by cardio-vascular and respiratory diseases is well documented [2]. Moreover, it has been demonstrated recently that air pollution plays a key role in increased susceptibility to viruses such as Severe Acute Respiratory Syndrome Coronavirus-2 (SARS-CoV-2) [3,4].

The skin is a portal to circulation and one of the main targets of pollutants since it is the body’s outermost barrier to the environment. Pollution aggressors induce biochemical and metabolic changes that cause premature skin aging, activate inflammatory pathways and generate oxidative stress responses that would ultimately affect dermal integrity and compromise overall skin health [5,6]. The United States Environmental Protection Agency shows that particulate matter (PM) is among the main air pollutants today [7] and 99% of the global population are exposed to fine PM pollution levels higher than the safe WHO guidelines (2.5 µg/m^3^) [1]. PM is a complex mixture formed mainly by a solid core and compounds such as polycyclic aromatic hydrocarbons (PAHs) and metals on the surface of the particles. Both metals and PAHs can affect tissue redox homeostasis thanks to their ability to induce oxidative stress by the formation of Reactive Oxygen Species (ROS), which could, in part, be generated from the particles themselves [8]. Consequently, to re-duce the excess of oxidative stress induced by PM exposure, the use of natural compounds with antioxidant properties is one of the main topical strategies to prevent cutaneous extrinsic aging [9].

The food-grade cyanobacterium *Spirulina* (*S. platensis*) is one of the most cultivated micro-algae worldwide [10]. The global *Spirulina* market is expected to reach close to USD 900 million by 2027 [11] and the increased demand for natural ingredients drives its anticipated market growth. Commonly commercialized as powder, capsules and tablets, *Spirulina* is used as an ingredient in food product formulations and manufacturing of food supplements. Its high concentration of protein, phytochemicals (phenolic compounds, carotenoids, chlorophylls, and others [12]) and related bioactivity [13,14,15] justify *Spirulina*’s status as a “superfood” by the WHO [16] and the rising interest in *Spirulina* by both the industry and academic sectors. 

*Spirulina* has also been explored for personal care and pharmaceutical uses such as cosmetics and skin health applications [17,18]. Topical applications developed with *Spirulina* extracts have efficiently improved epidermis structure [19], promoted faster wound healing and enhanced collagen production [20]. In tandem, there is solid evidence regarding the health protective effects of naturally occurring fruit-derived polyphenols [21,22,23]. In fact, because polyphenols are secondary metabolites found in large concentrations in fruit peels and seeds, fruit pomaces constitute a sustainable source of biomolecules for multiple skincare applications [24] and other health-related uses [25]. 

Microencapsulation is a technique that creates structured particles in which compounds of interest (core) are coated or embedded into polymeric materials (wall) that create a physical barrier against harmful surrounding conditions and thermal or oxidative-induced reactions. This delivery strategy is commonly applied to the stabilization of natural bioactive molecules for the formulation of food and pharmaceutical products [26,27,28] including ingredients for skincare formulations with enhanced properties and improved stability [29,30,31]. Spray drying microencapsulation is one of the most popular encapsulation techniques, because of its relatively low cost, easy operation, and suitability for heat-sensitive compounds. Most importantly, from a commercial standpoint, micro-encapsulated products can be tailored into premium ingredients with higher solubility, better taste, controlled release and easier incorporation into systems and products [32]. 

In this project, we introduced a new food-beauty approach where we used *Spirulina* protein as the wall material for spray drying microencapsulation of phytochemicals from berry pomaces to develop unique, clean label particles for skin health applications. Our hypothesis is that we can establish an efficient cosmeceutical production route by creating a synergistic combination of fruit pomaces and *Spirulina* phytochemicals for topical ap-plications to reduce pollutant penetration, improve skin barrier function and mitigate pollution skin damage. Initially, we evaluated the efficiency of the spray drying process to produce particles constituted by *Spirulina* protein and polyphenols recovered from elderberry and muscadine grape pomaces and characterized the resultant particles regarding their physicochemical characteristics (water activity and color) and phytochemical content (polyphenol load and phycocyanin content). Then, we prioritized a protein-polyphenol treatment for further assessment of its ability to mitigate pollution-induced skin inflammatory responses using ex vivo human biopsies exposed to diesel engine exhaust (DEE), a complex mixture of gases and particulates. To the best of our knowledge, this is the first report investigating skin health applications of spray dried microparticles produced by the complexation of repurposed polyphenol compounds recovered from side streams of the fruit industry and algae protein concentrate. Our study is aligned with the emerging food–beauty convergence world trend and unveils the potential benefits of using natural, good-enough-to-eat food resources as the primary ingredients for topical skincare products.

## 2. Materials and Methods

### 2.1. Materials

All organic solvents were analytical graded and obtained from VWR International (Suwanee, GA, USA). Organic *Spirulina* protein concentrate (SP, 66% protein, Zazzee Naturals, Austin, TX, USA) and xanthan gum (MakingCosmetics, Snoqualmie, WA, USA) were used in this study.

The elderberry (*Sambucus nigra*) pomace was obtained from Artemis International (Fort Wayne, IN, USA) and muscadine grape (*Vitis rotundifolia*) pomace originated from Noble variety grapes and was donated by Muscadine Grape Products (Wray, GA, USA) as side streams of the fruit processing industry. In both cases, they consisted of residual pulp, skins and seeds. 

### 2.2. Production of Concentrated Fruit Pomace Extracts

Initially, dry elderberry and muscadine pomaces were used to prepare concentrated fruit pomace extracts using a modified protocol based on our well-established method [33]. The elderberry pomace was mixed using a domestic blender (Ninja, Needham, MA, USA) with 50% ethanol solution and a extraction ratio of 1:3 (pomace:extraction solution, *w*/*v*). The ethanolic pomace solution was transferred to a laboratory water bath at 80 °C for 2 h, and filtered under vacuum using double layer cheese cloth, followed by centrifugation (ThermoFisher Scientific, Waltham, MA, USA) at 4000 rpm for 20 min. Finally, the ethanol was evaporated using a rotary evaporator in a water bath at 40 °C (Buchi Labortechnik AG, Flawil, Switzerland). Muscadine grape pomace extract was prepared by a similar protocol but using an extraction ratio 1:5 (pomace:extraction solution, *w*/*v*). The final concentrated elderberry pomace extract (EB) and muscadine grape pomace extract (MG) were kept under refrigeration (4 °C) until further use. 

### 2.3. Production of Spray Dried Spirulina Protein-Polyphenol Particles 

Solutions were prepared by blending *Spirulina* protein (SP) and fruit pomace concentrated extract at a ratio of 10% (*w*/*v*) and used for all experiments. Before spray drying, SP was added to the liquid fruit pomace concentrated extract and the mixture was homogenized for 2 h at room temperature using a magnetic stirrer to allow complete hydration. Two experimental *Spirulina* protein-polyphenol treatments consisting of elderberry pomace extract (EB) or muscadine grape pomace extract (MG) with SP were produced: SP-EB (*Spirulina* protein with elderberry pomace extract) and SP-MG (*Spirulina* protein with muscadine grape pomace extract).

Previously established protocols were used to produce spray-dried *Spirulina* protein- polyphenol particles [34]. The feed solution was atomized using a spray dryer (B-290, Buchi Labortechnik AG, Flawil, Switzerland) at 150 °C (outlet temperature 75–80 °C) following preliminary experiments. The spray drying system was operated using air in co-current flow and the following optimized conditions: 0.7 mm nozzle, 10 mL/min of feed flow (controlled by peristaltic pump), and 100% aspirator rate. The feed solution (concentrated fruit extract mixed with SP) was kept under constant magnetic stirring at 30 °C. The resulting spray dried (SD) *Spirulina* protein-polyphenol particles were collected from the collection chamber only, weighed, immediately sealed and kept frozen at −20 °C until further use. At least two independent batches of each treatment were prepared for each group. 

### 2.4. Percentual Solids Recovery and Polyphenol Retention 

The solids recovery of spray dried *Spirulina* protein-polyphenol samples was calculated as percentage (%) according to the ratio [total solids content of resulting particles (*Spirulina* protein-polyphenol particles)/total solids content in the feed solution (before spray drying)] × 100 according to our previous protocol [35]. The polyphenol retention was determined, as described by Grace et al. [34], as the percentage of polyphenols in the final spray dried *Spirulina* protein-polyphenol particles compared to the initial total polyphenol content in the feed solution, before spray drying.

### 2.5. Water Activity and Color Measurements 

The water activity of spray dried particles was measured using an Aqualab water activity meter (Decagon, Pullman, WA, USA). A reflectance spectrophotometer (CR-400, Konica Minolta, Tokyo, Japan), previously calibrated with white and black standards, was used to determine the color parameters lightness (L*), greenness (−a*) or redness (+a*), and blueness (−b*) or yellowness (+b*).

### 2.6. Phytochemical Analyses of Spray dried Spirulina Protein-Polyphenol Particles

Initially, 100 mg of SP and spray dried *Spirulina* protein-polyphenol particles were extracted twice, in an ultrasonic bath with 1 mL MeOH (1% Formic acid) for 10 min, centrifuged and supernatants collected. The pellet was extracted another time with 2 mL 80% MeOH to a total volume of 4 mL. The eluted extract was used for total phenolic assay, anthocyanin, proanthocyanin determinations, LCMS compound identification and antioxidant activity. 

#### 2.6.1. Total Phenolic Content (TPC)

The spectrophotometric quantification of total phenolics was performed by an adapted Folin–Ciocalteau method using microplates [36] at a wavelength of 765 nm (Spectramax Plus 384, Molecular Devices, Sunnyvale, CA, USA). Results were standardized against a gallic acid standard curve and expressed as gallic acid equivalent (mg GAE/g DW). 

#### 2.6.2. Anthocyanin Determination 

Anthocyanins (ANC) were analyzed using an Agilent 1260 series HPLC (Agilent Technologies, Santa Clara, CA, USA) equipped with a photodiode array detector (DAD). Samples were filtered through a 0.2 µm PTFE syringe filter (Fisher Scientific, Fair Lawn, NJ, USA) and separation was performed using an RP Supelcosil-LC-18 column, 250 mm × 4.6 mm × 5 μm (Supelco, Bellefonte, PA, USA). The mobile phase consisted of 5% formic acid in H_2_O (A) and 100% methanol (B). The flow rate was 1 mL/min with a step gradient of 10, 15, 20, 25, 30, 60, 100, 100, 10 and 10% of solvent B at 0, 5, 15, 20, 25, 46, 50, 50, 57 and 60 min, respectively, at constant temperature (30 °C). The results were expressed in mg cyanidin-3-*O*-glucoside equivalent/g DW established from a standard curve built with concentrations between 2–175 µg/µL.

#### 2.6.3. Proanthocyanidin Determination 

The total proanthocyanidin content (PAC) of SP and spray dried *Spirulina* protein-polyphenol particles was determined by an adapted DMAC assay using a calibration curve of procyanidin-B2 (3.125–100 µg/mL) to express results as mg procyanidin B2 equivalent/g DW [37]. The degree of polymerization of PAC compounds was determined according to our previous protocol [35] based on proanthocyanidin separation performed by an Agilent 1200 HPLC with fluorescence detector (HPLC-FLD), and a Develosil Diol normal phase column (250 mm × 4.6 mm × 5 μm, Phenomenex, Torrance, CA, USA). The binary mobile phase consisted of (A) acetonitrile/acetic acid (98:2, *v*/*v*) and (B) methanol/water/acetic acid (95:3:2, *v*/*v*/*v*). The flow rate was 0.8 mL/min with linear gradient at 35 °C, as follows: 0–35 min, 0–40% B; 35–40 min, 40–100% B; isocratic 100% B, 45 min; 100–0% B, 50 min; and 0% B to 55 min. Fluorescence detection was set at 230 nm excitation and 321 nm emission. PAC components with different degrees of polymerization (DP1, DP2, DP3 to DP6) were identified and quantified with a calibration curve of procyanidin-B2 (0.1–1.0 mg/mL).

#### 2.6.4. Carotenoid Extraction and Determination

For the carotenoid extraction [38], 40 mg of SP and spray dried *Spirulina* protein-polyphenol particles were incubated with 9 mL of ethanol (0.01% butylated hydroxytoluene, BHT) for 20 min at 40 °C, and then placed on ice where 3 mL of water was added, followed by 3 mL of hexane (0.01% BHT). The prepared solution was centrifuged (3000 rpm, 20 min at 10 °C) and the protocol was repeated twice. The collected extracts were pooled and dried over anhydrous sodium sulfate, decanted and supernatant was evaporated under nitrogen gas. Sample residue was re-suspended in 1 mL of extraction solution, filtered (0.2 µm PTFE) and 5 μL was injected immediately into HPLC-DAD (Agilent Infinity 1260 HPLC system, Agilent Technology, Santa Clara, CA, USA) and an autosampler (4 °C). Carotenoids and chlorophylls were separated using a reversed phase C30 column (250 × 4.6 mm × 5 μm, YMC America, Inc. Allentown, PA, USA). The solvents used were A: 85% methanol, 15% methyl-tert-butyl ether (MTBE) with 0.025% ammonium acetate and B: 10% methanol, 90% MTBE with 0.025% ammonium acetate. The solvent gradient was 10% B (5 min), 65% B (20 min), 95% B (15 min) and continued for 5 min, then back to 10% B (10 min), a flow rate of 1.5 mL/min. Compounds were monitored at 450 and 650 nm. Zeaxanthin, chlorophyll a and β-carotene standard references were used to build standard curves. Unidentified peaks were calculated as zeaxanthin equivalent. Triplicate samples were analyzed.

#### 2.6.5. Chlorophyll Determination

Samples of appropriate concentration (1.0 to 4.0 mg/mL) were mixed with 100% methanol, and centrifuged (4000 rpm, 10 min) to remove possible cell debris. The absorbance of supernatant was measured at 653 and 666 nm against a solvent blank using a UV spectrophotometer. Chlorophyll a and b contents were calculated by Equations (1) and (2) as mg/L where A_666_ and A_653_ are the absorbance values at 666 nm and 653 nm, respectively. Results were expressed as mg/g considering dilution factor in mg/g dry weight [39].

Chlorophyll a (mg/L) = 15.65 A_666_ − 7.34 A_653_ (1)

Chlorophyll b (mg/L) = 27.05 A_653_ − 11.21A_666_ (2)

#### 2.6.6. Phycocyanin Extraction and Determination

The extraction of phycocyanins (C-phycocyanins, CPC and Allophycocyanins, APC) was conducted according to a modified protocol [40]. Briefly, 50 mg of SP, or spray dried *Spirulina* protein-polyphenol particles were weighed in 15 mL centrifuge tubes. A potassium phosphate buffer (100 mM, pH 6.8) containing 0.06% sodium azide (10 mL) was added and tubes were vortexed and kept under refrigeration overnight. After that, samples were homogenized and centrifuged (15,000 rpm, 15 min at 4 °C). The supernatant was filtered (0.22 µm cellulose acetate filter) and the absorbance of each sample (620 nm and 652 nm) were read to determine CPC and APC concentrations following Equations (3) and (4) [41]:

CPC (mg/mL) = [OD_615_ − (0.474 × OD_652_)]/5.34 (3)

APC (mg/mL) = [OD_652_ − (0.208 × OD_615_)]/5.09 (4)

where OD_615_ is the optical density of the sample at 615 nm and OD_652_ is the optical density of the sample at 652 nm.

#### 2.6.7. Phenolic Composition by LC-ESI-TOF MS/MS 

It was determined using LC–MS (Shimadzu LC-IT-TOF-MS, Shimadzu, Tokyo, Japan) with Shim-pack XR-ODS column 2.2 μm (3.0 mm, 50 mm, Shimadzu, Japan). The solvent gradient consisted of 0.5% formic acid in water (A) and acetonitrile (B). Compounds were eluted into the ESI ion source at the flow rate of 0.3 mL/min with isocratic elution 5% B (0–15 min), then gradient 15–25% (15–25 min), 35% B (25–35 min), 95% B (35–40 min), isocratic 95% B (40–43 min). The column was re-equilibrated with 5% B for 5 min. The column was maintained at 40 °C during the run. The MS was programmed to carry out at full scan over a range of m/z 100–1100 (MS1) and m/z 100–700 (MS/MS) in both positive and negative ESI modes. The heat block and curved desolvation line (CDL) temperature was maintained at 200 °C. Nebulizing nitrogen gas was used at a flow rate of 1.5 L/min, and as the drying gas at 75 kPa. Phenolic compounds were characterized by their MS, MS/MS spectra, UV spectra, and in comparison with previously published data [42,43,44]. 

### 2.7. In Vitro Antioxidant Activity

#### 2.7.1. Free Radical Scavenging Activity 

The antioxidant capacity of the SP and *Spirulina*-polyphenol particles via DPPH^•^ (2,2-diphenyl-1-(2,4,6-trinitrophenyl)-hydrazinyl method was determined according to a previous report [45]. Briefly, samples (100 μL) were mixed with 3.9 mL of freshly prepared DPPH solution (0.08 M in 95% ethanol). After 40 min at room temperature, the absorbance was read at 515 nm against blak (Spectramax Plus 384, Molecular Devices, Sunnyvale, CA, USA). The standard curve was prepared using Trolox at μmol concentrations and results were expressed in μmol Trolox equivalent (TE)/g DW.

#### 2.7.2. Ferric Reducing Antioxidant Power (FRAP)

The antioxidant capacity of samples was estimated by the iron reduction method (FRAP), which is based on the ability of an antioxidant to reduce Fe^3+^ to Fe^2+^. It was conducted according to a previous report using ferrous sulfate as the standard reference [45]. The antioxidant capacity results were expressed in mmol ferrous sulfate equivalent/g DW.

### 2.8. Investigation of Pollution-Induced Skin Inflammatory Responses Using Ex Vivo Human Biopsies

#### 2.8.1. Preparation of Gel Samples Formulated with *Spirulina*-Fruit Pomace Particles

Initially, an aqueous solution containing 0.25% (*w*/*v*) of xanthan gum was prepared by mixing continuously the dried powder in deionized water on a magnetic stir plate for 30 min until complete dissolution. Then, xanthan-hydrocolloid solutions containing 100 µg/mL of SP particles only, MG particles only, and SP-MG particles (selected in the first part of this study) were prepared for the following experiments on the skin tissues.

#### 2.8.2. Ex Vivo Human BIOPSIES treatment and Exposure to Diesel Engine Exhaust (DEE)

Healthy human skin from three different donors was purchased from a local hospital (Hunstad/Kortesis/Bharti Cosmetic Surgery clinic, Huntersville, NC, USA; because the skin was purchased as “consumable material”, IRB and ethical approval was not required). After mechanical removal of excess subcutaneous fat with a scalpel, 20 skin biopsies (12 mm diameter) were obtained from each skin sample. After washing with phosphate-buffered saline (PBS) containing antibiotic-antimycotic, skin explants were maintained at 37 °C in a humidified 5% CO_2_ incubator in DMEM supplemented with 10% FBS, 100 U/mL penicillin, and 100 ug/mL streptomycin. The next day, the culture medium was replaced, and the SP, MG and SP-MG gels were topically applied. Then, 24 hours after pretreatment, skin explants were exposed for 30 min to DEE generated by a Kubota RTV-X900 diesel engine (3-cylinder, 4-cycle diesel with overhead valves, 1123 cc that has 24.8 HP at 3000 rpm). Skin biopsies were fixed overnight in 10% neutral-buffered formalin either immediately after the end of DEE exposure (T0) or after 24 h (T24).

#### 2.8.3. Immunofluorescence

Formalin-fixed skin explants were embedded in paraffin blocks, sectioned at 4 µm using a microtome and mounted on positive-charged slides. After xylene deparaffinization, skin sections were rehydrated in a decreasing gradient of ethanol. Then, heat-induced epitope retrieval was performed by immersing the skin sections in a sodium citrate buffer (ThermoFisher Scientific, Waltham, MA, USA) (pH 6.0) at a sub-boiling temperature in a 500 W microwave for 10 min. After blocking in 5% BSA for 30 min, skin sections were incubated overnight at 4 °C with the following primary antibodies diluted in PBS with 2% BSA: 4HNE (dil. 1:400; AB5605, Millipore Sigma, Burlington, MA, USA), COX-2 (dil. 1:400; NB100-868, Novus Biologicals, LLC, Centennial, CO, USA), involucrin (dil. 1:50; sc-21748, Santa Cruz Biotechnology, Inc., Dallas, TX, USA). Slides were then incubated at RT for 1h in the dark with fluorochrome-conjugated secondary antibodies diluted in 2% BSA (dil. 1:1000) (A11004, Alexa Fluor 568 and A11055, Alexa Fluor 488, ThermoFisher Scientific Inc.). After nuclear staining with DAPI (D1306, ThermoFisher Scientific Inc.), sections were mounted using PermaFluor mounting medium (TA006FM, ThermoFisher Scientific Inc.). Images were acquired using a Zeiss LSM 710 microscope (Carl Zeiss, Thornwood, NY, USA) and Zen 2008 Software (Carl Zeiss Microscopy GmbH, Jena, Germany) with a 40× objective. Images were quantified using an open-source image analysis software (ImageJ 1.52a) [46].

### 2.9. Statistics

Statistical analyses were performed using GraphPad Prism 6 software (GraphPad Software Inc., La Jolla, CA, USA). For comparisons between groups, analysis of variance (ANOVA) followed by Tukey’s post hoc test was conducted using *p* ≤ 0.05 significance level, unless noted. All data were expressed as mean ± standard deviation. 

## 3. Results

### 3.1. Spray Drying Evaluation

**Figure 1:**
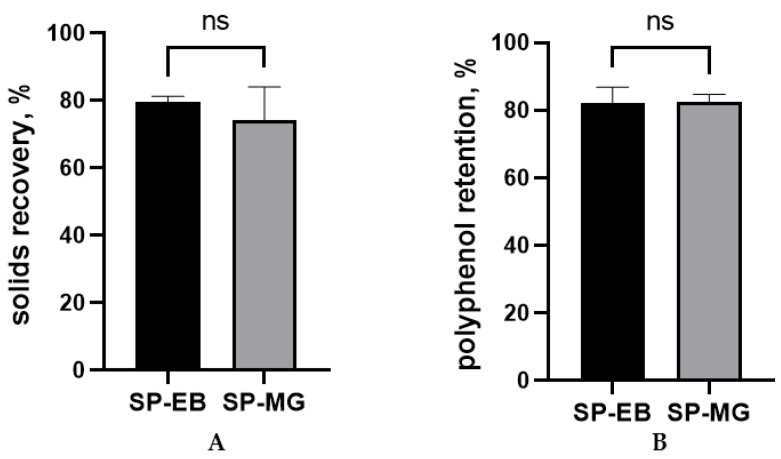
Solids recovery (**A**) and polyphenol retention (**B**) for the spray drying microencapsulation of polyphenols from berry pomace extracts with *Spirulina* protein. SP-EB: spray dried *Spirulina* protein and elderberry pomace extract and SP-MG: *Spirulina* protein and muscadine grape pomace extract. ns: not statistically significant, *p* > 0.05.

In this study, similar solids recovery and polyphenol retention were observed for spray dried protein-polyphenol particles produced with elderberry and muscadine grape pomace extracts (*p* > 0.05; Figure 1). In fact, both treatments SP-EB and SP-MG achieved remarkable results (approximately 80%) for both parameters, considerably higher than the 50% threshold considered as satisfactory for lab-scale spray drying processes [47]. 

### 3.2. Characterization of Spray-Dried Spirulina Protein-Polyphenol Particles

#### 3.2.1. Physicochemical Evaluation

**Figure 2:**
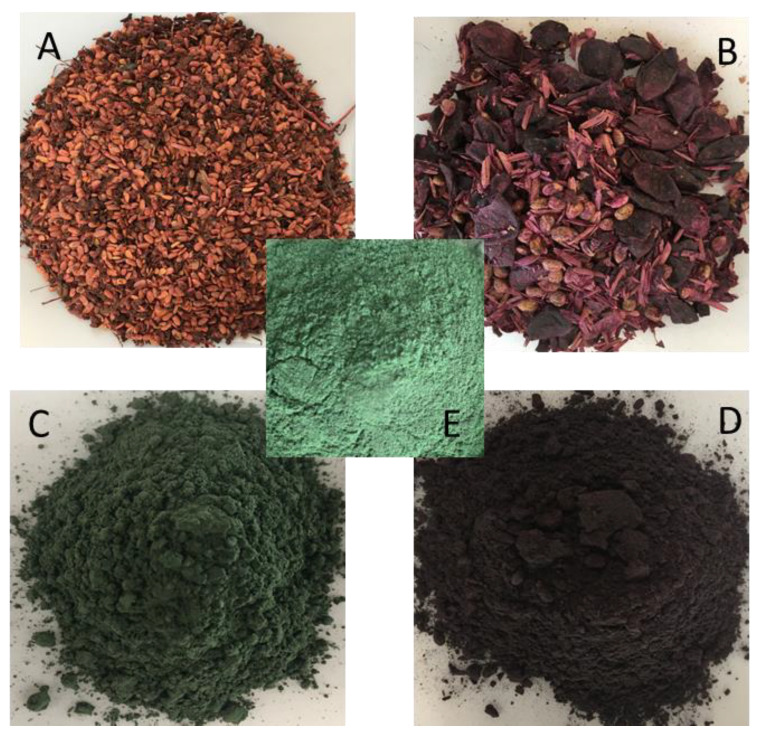
(**A**) Elderberry pomace and (**B**) Muscadine grape pomace used in this study and spray dried *Spirulina* protein-polyphenol particles produced by the complexation of *Spirulina* protein and polyphenols extracted from elderberry and muscadine grape pomaces: (**C**) SP-EB, spray dried *Spirulina* protein and elderberry pomace extract and (**D**) SP-MG, spray dried *Spirulina* protein and muscadine grape pomace extract. (**E**) *Spirulina* protein concentrate.

Figure 2 shows the materials used in this study. Both elderberry and muscadine grape pomaces (Figure 2A,B) are constituted by seeds, peels and residual fruit pulp. The remarkable differences observed for the CIELAB color parameters (Table 1) reflect the different color patterns observed visually. Spray dried SP-EB particles (Figure 2C) showed a dark green color that resembles the original green color of *Spirulina* protein (Figure 2E), while SP-MG particles (Figure 2D) had an intense purple color typical of Noble muscadine grapes used in this study. 

Water activity (Aw) measures the available free water that could potentially induce or facilitate deteriorative reactions, and therefore it is an important index to estimate the shelf life of particulate products. The spray dried particles had similar (*p* > 0.05) water activity levels (Table 1), within the typical range for spray dried powders [36] and within the microbiologically stable range [48].

#### 3.2.2. Phytochemical Analysis

Results of quantitative analysis for phenolics, carotenoids, chlorophylls and phycocyanins, all contributing to the antioxidant activity of the formulated particles, are reported in Table 2. Total phenolic content measured by Folin Ciocalteu assay indicated that SP (no added phenolics) had a low TPC of 7.43 ± 0.23 mg GAE/g DW, but when complexed with phenolic-rich pomace extracts obtained from elderberry or muscadine grape pomace, a substantial increase was observed, reaching 27.63 ± 1.15 and 114.27 ± 6.17 mg GAE/g DW for SP-EB and SP-MG particles, respectively. 

The *Spirulina* protein-polyphenol particles showed a total anthocyanins content of 2.76 ± 0.25 and 6.52 ± 0.46 mg cyanidin-3-*O*-glucoside equivalent/g DW for SP-EB and SP-MG, respectively. The HPLC profiles showed typical chromatograms for anthocyanins present in elderberry [49] and muscadine grapes [50]. The identification of anthocyanins was confirmed by LC-MS/MS (Table A1). EB had mono and diglucosides of cyanidin, while MG anthocyanins comprised diglucosides belonging to diverse anthocyanidins: delphinidin, cyanidin, petunidin, peonidin and malvidin. 

**Figure 3:**
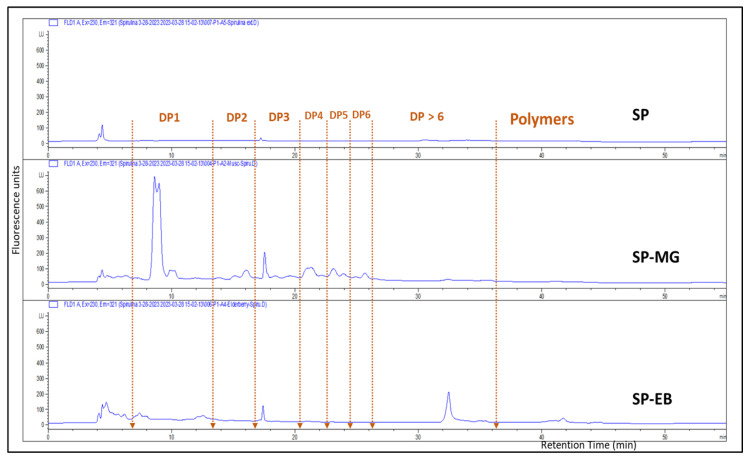
HPLC-FLD chromatograms of *Spirulina* protein (SP), spray dried *Spirulina* protein and elderberry pomace extract (SP-EB) and *Spirulina* protein and muscadine grape pomace extract (SP-MG).

Another flavonoid group of compounds, named flavan-3-ols or proanthocyanins (PAC), were present in higher concentration in SP-MG compared with SP-EB particles. HPLC-fluorescence detection measurements, based on peak area against procyanidin-B2 standard curve, showed 2.65 ± 0.12 and 9.02 ± 0.74 mg/g DW for SP-EB and SP-MG, respectively (Figure 3). LCMS identified 18 flavan-3-ols, (proanthocyanidins, or condensed tannins) in SP-MG (Table A1). A series of monomers, dimers and higher conjugations of PAC, in addition to their sugar and gallic acid conjugations were found in SP-MG particles. LC-MS/MS indicated that SP-MG particles had a total of 45 identified compounds with structurally diverse phenolic composition. 

Carotenoids, chlorophylls, and phycocyanins are pigments present in *Spirulina* algae, and contribute to the antioxidant activity of *Spirulina* protein-polyphenol particles. β-carotene was the major identified carotenoid in SP. Total carotenoids totaled 3.10 ± 0.06, 3.44 ± 0.17 and 2.44 ± 0.23 mg/g DW for SP, SP-EB and SP-MG, respectively. The higher carotenoid content of SP-EB compared to SP is a contribution from elderberry pomace extract [51]. Additionally, *Spirulina* is a great source of chlorophylls, specifically chlorophyll a, a phytochemical with detoxifying and purifying activity [16]. Chlorophyll a measured 15.24 ± 0.76, 13.93 ± 0.26 and 6.26 ± 0.47 mg/g DW while chlorophyll b recorded 4.07 ± 0.22, 4.23 ± 0.19, 2.53 ± 0.18, for SP, SP-EB and SP-MG, respectively. *Spirulina* is one of the greatest dietary sources of C-phycocyanin, a water-soluble protein pigment with potent antioxidant activity [52]. C-phycocyanins (C-PC) measured 443.2 ± 1.10, 257 ± 0.70, and 71.95 ± 0.53 mg/g DW, in addition to another phycocyanin, allophycocyaninn APC, contributed an amount of 246.2 ± 2.2, 136 ± 0.47, and 42.75 ± 1.55 mg/g DW for SP, SP-EB and SP-MG, respectively. 

Both antioxidant assays (DPPH and FRAP) confirmed the higher antioxidant activity of SP-MG particles compared to SP-EB. A DPPH radical scavenging assay showed 120 ± 11.0 and 70.7 μM TE/g DW for SP-MG and SP-EB, respectively, while an FRAP assay detected 203.4 ± 23.4 µM Fe^+2^ E/g DW for SP-MG and 61.00 ± 6.85 µM Fe^+2^ E/g DW for SP-EB, as a consequence of the higher TPC, proanthocyanidin and anthocyain contents of SP-MG particles (Table 2).

### 3.3. Evaluation of Pollution-Induced Skin Damage

**Figure 4:**
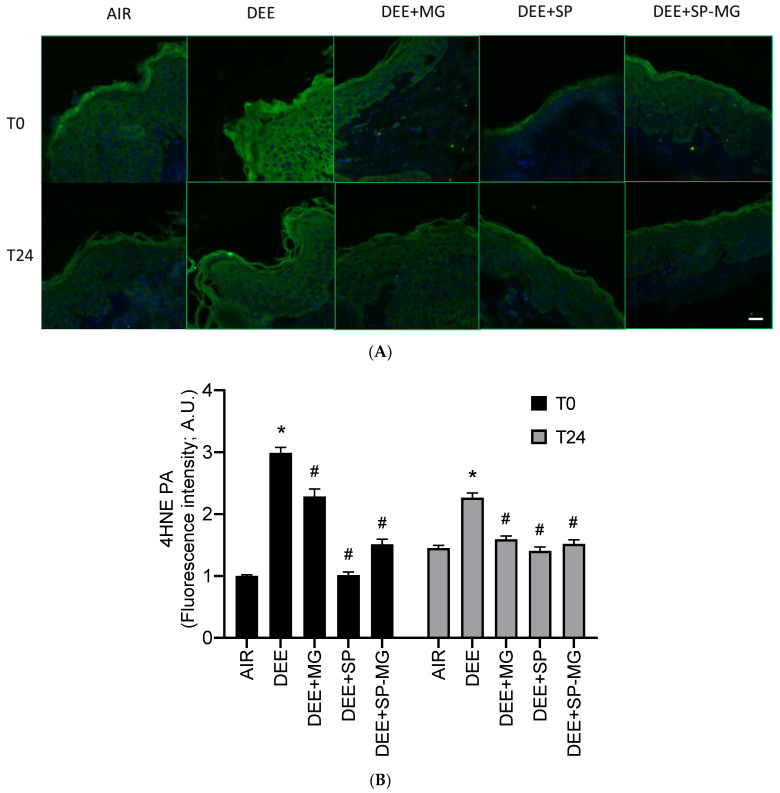
Topical application of gel formulated with spray dried *Spirulina* protein-polyphenol particles (SP-MG, SP complexed to polyphenols from muscadine grape pomace extract) mitigates protein oxidative damage in skin exposed to DEE. (**A**) Representative confocal images of 4HNE-PA staining (green) in skin biopsies untreated or pre-treated with muscadine grape extract (MG), SP or SP-MG gel and then, exposed to DEE. Nuclei are stained with DAPI (blue). Original magnification 40×. Scale bar 20 μm. (**B**) Semi-quantification of the immunofluorescence intensity performed by ImageJ is shown in the graph. Data are expressed as arbitrary units (A.U., average of three independent experiments), * *p* ≤ 0.0001 Air vs. DEE; # *p* ≤ 0.0001 DEE vs. DEE + MG, DEE + SP or DEE + SP-MG by ANOVA within the same time point (T0 or T24).

Traffic-related air pollution is a major health risk factor and is strongly associated with human pathological conditions [53,54]. In the cutaneous tissue, exposure to vehicle exhaust from diesel impairs skin barrier function by promoting oxidative damage and subclinical inflammatory responses [55,56]. Accordingly, in our study, skin biopsies exposed to DEE showed a strong immunoreactivity for 4-hydroxynonenal protein adducts (4HNE-PA), a well-known marker of lipid peroxidation-mediated protein damage, in the epidermis and, particularly, in its outermost layer, the stratum corneum (Figure 4A). Levels of 4HNE-PA significantly increased at the end of DEE exposure (T0) and, albeit with a decreasing trend, persisted higher in DEE-exposed skin biopsies than in unexposed tissues for up to 24 h (Figure 4B). Furthermore, analysis of immunofluorescence signal intensity indicated that topical pretreatment with SP gel and, to a greater extent, MG and SP-MG gels mitigated the formation of 4HNE-PA in DEE-exposed skin tissues both at T0 and T24 (Figure 4B).

**Figure 5:**
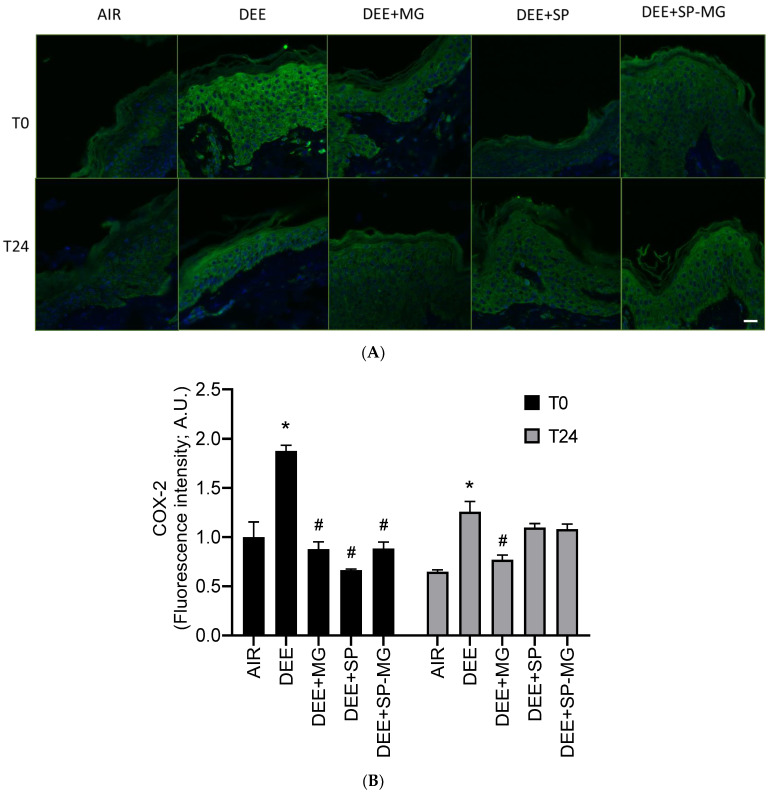
Topical application of gel formulated with spray dried *Spirulina* protein-polyphenol particles (SP-MG, SP complexed to polyphenols from muscadine grape pomace extract) moderates the proinflammatory response in skin exposed to DEE. (**A**) Representative confocal images of COX-2 staining (green) in skin biopsies untreated or pre-treated with muscadine grape extract (MG), SP or SP-MG gel and then, exposed to DEE. Nuclei are stained with DAPI (blue). Original magnification 40×. Scale bar 20 μm. (**B**) Semi-quantification of the immunofluorescence intensity performed by ImageJ is shown in the graph. Data are expressed as arbitrary units (A.U., average of three independent experiments), * *p* ≤ 0.0001 Air vs. DEE; # *p* ≤ 0.0001 DEE vs. DEE + MG, DEE + SP or DEE + SP-MG by ANOVA within the same time point (T0 or T24).

Pollution-mediated oxidative stress and damage can in turn activate inflammatory signaling pathways in a crosstalk known as oxinflammation [56]. In particular, 4HNE is a well-known inducer of cyclooxygenase-2 (COX-2), the rate-limiting enzyme for the synthesis of arachidonic acid-derived prostanoids, i.e., lipid mediators that regulate the inflammatory response [57]. Similarly to 4HNE-PA formation, a strong green immunofluorescence signal for COX-2 was evident in the epidermis and dermis after DEE exposure, while pretreatment with SP, MG and SP-MG gels counteracted this effect (Figure 5A). By quantifying the fluorescence signal, we observed that the COX-2 expression significantly increased 30 min after DEE exposure (T0) and remained at higher levels than that in unexposed tissues up to 24 h (Figure 5B). At T0, DEE-induced COX-2 overexpression was completely prevented by a 24 h pretreatment with the three particle gels and again mitigated by the MG gel, 24 h after the end of exposure (Figure 5B). 

**Figure 6:**
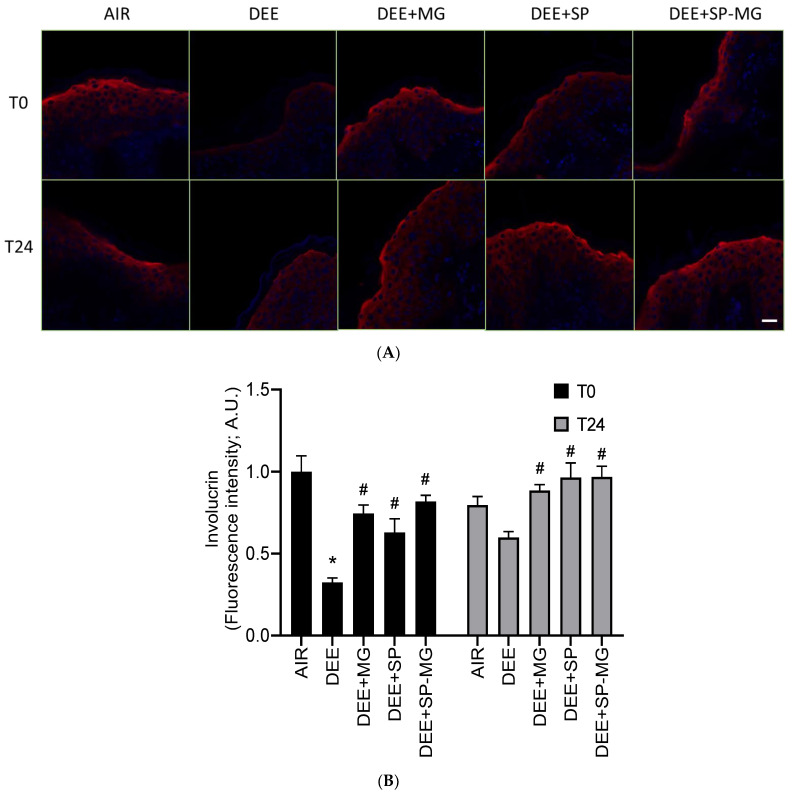
Topical application of gel formulated with spray dried *Spirulina* protein-polyphenol particles (SP-MG, SP complexed to polyphenols from muscadine grape pomace extract) prevents the loss of involucrin in skin exposed to DEE. (**A**) Representative confocal images of involucrin staining (red) in skin biopsies untreated or pre-treated with muscadine grape extract (MG), SP or SP-MG gel and then, exposed to DEE. Nuclei are stained with DAPI (blue). Original magnification 40×. Scale bar 20 μm. (**B**) Semi-quantification of the immunofluorescence intensity performed by ImageJ is shown in the graph. Data are expressed as arbitrary units (averages of three independent experiments), * *p* ≤ 0.0001 Air vs. DEE; # *p* ≤ 0.0001 DEE vs. DEE + MG, DEE + SP or DEE + SP-MG by ANOVA within the same time point (T0 or T24).

Oxidative and inflammatory signaling cascades triggered by pollutants negatively impact the structure and function of the cutaneous tissue, compromising skin barrier integrity [56,58]. Consistent with previous reports, exposure of skin tissue to DEE clearly decreased the red immunofluorescence signal for involucrin, a structural protein component of the cornified cell envelope, whereas this effect was avoided by pretreatment with the three gels (Figure 6A). In particular, the levels of involucrin markedly decreased 30 min after exposure to DEE and showed to be partially restored 24 h after the end of exposure (Figure 6B). Notably, pretreatment with SP, MG and SP-MG gels moderated, to a similar extent for all three gels, the pollutant-mediated decline in involucrin expression at T0 and restored it to the physiological level at T24 (Figure 6B).

## 4. Discussion

### 4.1. Assessment of Spray Dried Spirulina Protein-Polyphenol Particles 

Both the solids recovery and polyphenol retention are key parameters in the evaluation of the spray drying performance. The solids recovery parameter is referred to as process yield, since it expresses the solids yield percentage by calculating the relationship between the spray dried solids recovered after drying and initially present in the feed solution [36]. Polyphenol retention measures the ability of carrying the polyphenols from the feed solution into the newly formed particles [59]. The higher the solids recovery and polyphenol retention are, the more efficient and feasible an SD process is. In our study, we showed that it is possible to efficiently produce *Spirulina* protein-polyphenol particles with both high solids recovery and polyphenol retention (approximately 80%). These findings indicate that *Spirulina* protein is an effective carrier to protect and deliver polyphenols recovered from both berry pomaces.

Spray drying microencapsulation of SP and polyphenols from berry pomaces produced particles with vibrant colors and water activity levels within the chemically stable range (Aw < 0.5). In fact, phytochemical-rich spray dried powders not only warrant the obtention of products with extended shelf-life by significantly reducing the moisture content and minimizing the risk of microbial spoilage (compared to slurries or liquid formats), but also warrants enhanced flowability, and easier handling and mixing with other ingredients, expanding manufacturing frameworks for the creation of complex blends for food, pharmaceuticals and cosmetic purposes [60].

*Spirulina* protein contributed carotenoids, chlorophylls and phycocyanin compounds to the complex composition of both SP-EB and SP-MG (Table 2). A remarkable concentration of phycocyanins were detected in SP used in this study and CPC was detected in higher amounts than APC (Table 2). CPC is a blue water-soluble phycobiliprotein pigment that has been successfully used for multiple applications in the cosmetics, pharmaceuticals and food industries [61]. The pigment composition of SP also included carotenoids (mainly all-trans β-carotene, 1.79 and cis β-carotene, 0.46 mg/g DW) and chlorophylls (mainly chlorophyll a, 15.24 mg/g DW). Similar β-carotene was reported in *Spirulina* algae cultivated in different media conditions [62].

Both elderberry and muscadine grape pomaces are remarkable bioresources of health-relevant phenolic antioxidants [63,64,65,66]. Indeed, our phytochemical analysis revealed that phenolic compounds are major bioactive molecules captured in the spray dried Spirulina protein-polyphenol particles (Table 2). SP-MG concentrated significantly higher phenolic content, with an outstanding concentration of proanthocyanidin compounds and significantly higher anthocyanins content compared to SP-EB. In addition to phenolic acids, anthocyanins and flavonols and their analogues which were present in EB-SP particles, SP-MG contained hydrolyzable tannins, which can be classified as gallotannins and ellagitannins. Gallotannins consist of a glucose molecule in which hydroxyl groups are partly or completely substituted with galloyl groups, and ellagitannins are esters of the hexahydroxydiphenoyl (HHDP) group consisting of a polyol core (glucose or quinic acid). These compounds are important due to their potential health benefits as antioxidants and their anticancer, anti-inflammatory, and cardioprotective activities [67]. The hydrolyzable tannins fraction from Poincianella pluviosa stem bark showed wound healing properties in vitro by increasing mitochondrial activity and a proliferation of keratinocytes and dermal fibroblasts [68]. Due to the redox characteristics that phenolics possess, they play a significant role as antioxidants and are very efficient in scavenging harmful free radicals. On average, 87.1%, 11.3%, and 1.6% of phenolic compounds are present in seeds, skin and pulp of the muscadine grape, respectively [69]. Muscadine grapes have significant flavonoid content retained in their leftover skins, peels and seeds after juicing, thus it is not surprising, as shown in our results, that the pomace is an excellent resource for valuable residual polyphenolic compounds. 

Due to the high polyphenol content and antioxidant activity measured by both DPPH and FRAP methods, and substantial amounts of carotenoids, chlorophylls and phycocyanins observed for SP-MG particles, this treatment was prioritized for the preparation of a topical gel formulation further assessed for its ability to mitigate pollution-induced skin oxinflammatory responses using ex vivo human biopsies exposed to diesel engine exhaust (DEE). 

### 4.2. Investigation of Pollution-Induced Skin Inflammatory Responses with and without Topical Pretreatment with Gel Formulated with SP-MG Particles

The rising interest for sustainable products made with repurposed ingredients has reached the cosmetic industry [24]. Cosmeceuticals are now the fastest growing segment of an emerging natural personal care industry [70]. However, the task of recovering bioactive compounds from by-products and repurposing them as active molecules for higher value applications is technologically challenging [71], due to the ephemeral nature of phytochemicals and the necessity of a careful definition of effective processing steps. 

In this study, we evaluated the protein-polyphenol complexation as a strategy to stabilize and deliver high-value natural phytoactives from berry pomaces. In particular, the muscadine grape pomace, a US southern staple, proved to be a natural resource with substantial amounts of residual phytochemicals that can be revalorized into value-added skin products [72]. 

Indeed, our results in ex vivo human skin biopsies clearly confirm the effectiveness of the topically applied SP-MG gel in mitigating the oxinflammatory phenomena that occur following exposure to diesel fumes. Emissions from on-road diesel vehicles are among the most predominant pollutants worldwide, responsible for serious health conditions affecting the respiratory, cardiovascular, endocrine, gastrointestinal, nervous and reproductive systems, among others [54]. At the interface with our external environment, the skin is a major target organ for the detrimental actions of DEE [56]. Diesel engine emissions consist of hundreds of toxic species in either solid, condensed or gaseous phase. By interacting with skin constituents or penetrating through the stratum corneum, DEE toxic compounds promote the generation of ROS and inflammatory mediators by activating a plethora of stress signaling responses, as shown here and in previous studies by increased protein oxidative damage (i.e., 4HNE-PA) and inflammation (i.e., COX-2) associated with skin barrier disruption (i.e., loss of involucrin) [58,73,74]. In addition to building healthy skin with a proper and balanced diet, the daily topical application of cosmeceuticals containing functional active ingredients represents a powerful tool in the treatment and prevention of pollution-induced premature aging [75]. 

In this context, our data support the use of SP-MG gel, a value-added skin product delivering residual fruit/algae phytoactives, for skin health applications aimed at mitigating pollution-related oxinflammation and preserve skin barrier integrity. Further investigations will clarify the underlying mechanisms of action which could include the direct antioxidant properties of SP-MG gel and/or the likely ability of SP-MG phytoactives in activating cellular defense systems such as, for example, those under the control of NRF2 [76].

## 5. Conclusions

Air pollution is the world’s major environmental health risk. Novel protection strategies would benefit mainly the large world’s population that live in urban areas, and therefore, more prone to be affected by pollution-associated skin damage. The incorporation of *Spirulina* protein-polyphenol particles into skin products directed for topical applications is a cosmeceutical strategy to reduce the load of potentially harmful compounds on the skin and improve skin barrier function, thus reducing pollutant penetration and damage to the skin. 

## Figures and Tables

**Table 1:** Water activity (Aw) and color parameters of spray dried *Spirulina* protein-polyphenol particles produced by the complexation of *Spirulina* protein and polyphenols extracted from elderberry and muscadine grape pomaces ^1^.

	SP-EB	SP-MG
L*	34.82 ± 0.35 ^a^	28.11 ± 0.47 ^b^
a*	−6.51 ± 0.29 ^a^	−1.13 ± 0.02 ^b^
b*	−0.65 ± 0.10 ^a^	−5.97 ± 0.08 ^b^
Aw	0.304 ± 0.005	0.262 ± 0.006

^1^ Spray dried *Spirulina* protein-polyphenol particles: SP-EB, *Spirulina* protein and elderberry pomace extract and SP-MG, *Spirulina* protein and muscadine grape pomace extract. Results are shown as mean ± standard deviation. Superscripts with different letters (a, b) in the same row are significantly different (*p* < 0.001). CIELAB parameters: L*: lightness; color coordinates: a*—green to red; b*—blue to yellow.

**Table 2:** Phytochemical composition and antioxidant activity for *Spirulina* protein and spray-dried *Spirulina* protein-polyphenol-rich particles ^1^.

	SP ^1^	SP-EB ^2^	SP-MG ^3^
**Phenolics** (mg/g DW)			
Total phenolics, as GAE ^4^	7.43 ± 0.23	27.63 ± 1.15	114.27 ± 6.17
Total proanthocyanidins, as PAC-B2 ^5^	--	2.65 ± 0.12	9.02 ± 0.74
Total anthocyanin, as C3G ^6^	--	11.39 ± 0.46	26.87 ± 1.25
**Carotenoids** (mg/g DW)			
Zeaxanthin	0.40 ± 0.04	0.28 ± 0.04	0.20 ± 0.04
Other xanthophylls (as zeaxanthin)	0. 45 ± 0.00	1.06 ± 0.14	0.85 ± 0.00
all-trans β-Carotene	1.79 ± 0.03	1.65 ± 0.08	1.11± 0.21
cis β-Carotene	0.46 ± 0.01	0.45 ± 0.03	0.28 ± 0.06
Total carotenoids	(3.10 ± 0.06)	(3.44 ± 0.17)	(2.44 ± 0.23)
**Chlorophylls** (mg/g DW)			
Chlorophyll a	15.24 ± 0.76	13.93 ± 0.26	6.26 ± 0.47
Chlorophyll b	4.07 ± 0.22	4.23 ± 0.19	2.53 ± 0.18
**Phycocyanins** (mg/g DW)			
C-Phycocyanins (CPC)	443.2 ± 1.10	257 ± 0.70	71.95 ± 0.53
Allo-phycocyanins (APC)	246.2 ± 2.2	136 ± 0.47	42.75 ± 1.55
**Antioxidant activity**			
DPPH assay (µM TE/g DW) ^7^	8.04 ± 0.70	70.7 ± 5.8	120 ± 11.0
FRAP assay (µM Fe^+2^ E/g DW) ^8^	31.88 ± 1.61	61.00 ± 6.85	203.4 ± 23.4

^1^ SP: *Spirulina* protein and spray dried *Spirulina* protein-polyphenol particles: ^2^ SP-EB *Spirulina* protein and elderberry pomace extract and ^3^ SP-MG *Spirulina* protein and muscadine grape pomace extract. ^4^ GAE: Gallic acid equivalent. ^5^ PAC-B2: proanthocyanidin-B2 equivalent. ^6^ C3G: cyanidin-3-*O*-glucoside equivalent. ^7^ TE: Trolox equivalent. ^8^ µM Fe^+2^ E: µmol ferrous sulfate equivalent. Results are shown as mean ± standard deviation (n = 3).

## Data Availability

Data are contained within this article.

## Appendix A

**Table A1:** Retention time, mass spectroscopic data of phenolic compounds identified in SP-EB and SP-MG particles.

*Spirulina*-Elderberry Particles			
RT (min)	*m*/*z* [M + H]^+^, (MS/MS)	*m*/*z* [M-H]¯, (MS/MS)	Tentative Identification
Phenolic acids			
1.39		367 [M-H]¯, (191, 173)	3-Feruloylquinic acid
1.64		341 [M-H]¯, (230)	Caffeic acid-hexoside
21.96	355 [M + H]^+^, (164, 154)		Chlorogenic acid
22.70	355 [M + H]^+^, (163, 145)		Neochlorogenic acid
23.35		515 [M-H]¯, (353, 191)	1,5-Di-O-caffeoylquinic acid
26.67		515 [M-H]¯, (253, 191)	3,4-Di-O-caffeoylquinic acid
27.36		515 [M-H]¯, (253, 191)	4,5-Di-O-caffeoylquinic acid
Anthocyanins			
21.86	611 [M]^+^, (449, 287)		Cyanidin-3,5-diglucoside
22.11	743 [M]^+^, (581, 449, 287)		Cyanidin-3-sambuboside-5-glucoside
23.12	449 [M]^+^, (287)		Cyanidin-3-glucoside
23.25	581 [M]^+^, (287)		Cyanidin-3-sambuboside
Flavonols			
23.41	595 [M + H]^+^, (449, 287)		Kaempferol-rutinoside
23.55		433 [M-H]¯, (271)	Quercetin-pentoside
25.83		609 [M-H]¯, (301)	Quercetin-rhmnoside-hexoside
25.87	611 [M + H]^+^ (303)		Quercetin-3-rutionside
26.08	465 [M + H]^+^, (303)		Quercetin-3-glucoside
26.78	596 [M + H]^+^, (449, 287)		Kaempferol-rutinoside
27.01		623 [M-H]¯, (315, 300)	Isorhmentin-rutinoside
27.30		477 [M-H]¯ (315)	Isorhmnetin-glucoside
33.10		447 [M-H]¯ (285)	Kaempferol-hexoside
Flavan-3-ols			
1.49		289 M-H]¯, (191)	Epicatechin
26.12		577 M-H]¯, (463, 301)	Procyanidin dimer B
***Spirulina*-muscadine grape particles**			
Phenolic acids			
1.10		179 [M-H]¯	Caffeic acid
1.38		195 [M-H]¯	Gluconic acid
23.34		311 [M-H]¯	Caftaric acid
Anthocyanins			
20.54	627 [M]^+^, (465, 303)		Delphinidin-3,5-diglucoside
21.11	611 [M]^+^, (449, 287)		Cyanidin-3,5-diglucoside
21.59	641 [M]^+^, (479, 317)		Petunidin-3,5-diglucoside
22.23	625 [M]^+^, (463, 301)		Peonidin-3,5-diglucoside
22.41	655 [M]^+^, (493, 331)		Malvidin-3,5-diglucoside
20.54	627 [M]^+^, (465, 303)		Delphinidin-3,5-diglucoside
21.10	611 [M]^+^, (449, 287)		Cyanidin-3,5-diglucoside
Flavonols			
20.495	465 [M + H]^+^, (317)		Myricetin rhamnoside
20.50	465 [M + H]^+^, (303)		Quercetin-hexoside
21.95	479 [M + H]^+^, (317)		Myricetin-hexoside (1)
22.17	479 [M + H]^+^, (317)		Myricetin-hexoside (2)
26.55	449 [M + H]^+^, (303)		Quercetin-rhamnoside
26.5	433 [M + H]^+^, (287)		Kaemperol rhamnoside (1)
27.66	433 [M + H]^+^, (287)		Kaemperol rhamnoside (2)
Flavan-3-ols and proanthocyanidins			
20.43		289 [M-H]¯, (245, 179, 161)	Catechin (Cat)
20.89		471 [M-H]¯, (331, 213)	O-Methyl-catechin gallate (1)
21.66		451 [M-H]¯, (331, 289, 245)	Catechin-hexoside (1)
22.19		451 [M-H]¯, (331, 289, 245)	Catatechin-hexoside (2)
22.47		577 [M-H]¯, (407, 289)	Procyanidin dimer B (1)
22.92		577 [M-H]¯, (407, 289)	Procyanidin dimer B (2)
23.34		289 [M-H]¯, (245, 179, 161)	Epicatechin
23.83		467 [M-H]¯, (305, 165)	Gallocatechin/Epigallocatechint-hexoside (1)
24.15		577 [M-H]¯, (407, 289)	Procyanidin dimer B (3)
24.39		467 [M-H]¯, (305, 165)	Gallo-Catechin/Epicatechin-hexoside (2)
24.96		441 [M-H]¯, (289)	Catechin/Epicatechin-gallate (1)
25.27		577 [M-H]¯, (407, 289)	Procyanidin dimer B (4)
25.32		441 [M-H]¯, (289)	Catechin/Epicatechin-gallate (2)
26.08		577 [M-H]¯, (407, 289)	Procyanidin dimer B (5)
26.53		471 [M-H]¯ (331, 213)	O-Methyl-Catechin/Epicatichin-gallate
27.13		441 [M-H]¯, (289)	Catechin/Epicatechin-gallate (3)
27.62		441 [M-H]¯, (289)	Catechin/Epicatechin-gallate (4)
28.53		451 [M-H]¯ (331, 289, 245)	Catechin/Epicatechin-hexoside (3)
Gallic acid and conjugates			
2.12		331 [M-H]¯, (271, 169)	Galloyl hexoside (1)
2.32		169 [M-H]¯, (125)	Gallic acid
5.35		331 [M-H]¯, (271, 169)	Galloyl-hexoside (2)
6.63		331 [M-H]¯, (271, 169)	Galloyl-hexoside (3)
28.52		493 [M-H]¯	Galloyl-di-hexoside (1)
30.62		493 [M-H]¯	Galloyl-di-hexoside (2)
Ellagic acid and conjugates (ellagitannins)			
20.47		625 [M-H]¯, (463, 301)	HHDP-diglucoside
21.90		633 [M-H]¯, (481, 301, 275)	HHDP-galloyl glucoside
24.97		433 [M-H]¯, (301)	Ellagic acid xyloside
25.25		463 [M-H]¯, (301)	Ellagic acid hexoside
25.33		301 [M-H]¯, (229, 185, 151)	Ellagic acid
28.63		447 [M-H]¯, (301)	Ellagic acid rhamnoside 1
30.62		447 [M-H]¯, (301)	Ellagic acid rhamnoside 2
